# Evaluation of hydrolyzed salmon and hydrolyzed poultry feather diets in restrictive diet trials for diagnosis of food allergies in pruritic dogs

**DOI:** 10.3389/fvets.2025.1560806

**Published:** 2025-05-08

**Authors:** Thomas P. Lewis, George E. Moore, Carine Laporte, Leighann Daristotle, Nolan Z. Frantz

**Affiliations:** ^1^Dermatology for Animals, Gilbert, AZ, United States; ^2^College of Veterinary Medicine, Purdue University, West Lafayette, IN, United States; ^3^Dermatology for Animals, Salt Lake City, UT, United States; ^4^Blue Buffalo Co. Ltd., Number One General Mills Blvd., Minneapolis, MN, United States

**Keywords:** cutaneous adverse food reaction, elimination diet trial, hypoallergenic food, food reaction, allergy, dog, canine

## Abstract

**Introduction:**

Canine cutaneous adverse food reaction (CAFR) is a common disorder caused by abnormal and unwanted skin reactions to ingested dietary allergens. Whereas other forms of allergic dermatitis may require drug therapy, CAFR is best treated with dietary change. Therefore, early and accurate diagnosis and treatment of CAFR are critical. The gold standard test for CAFR is a 6–12 week elimination diet trial using limited and known hypoallergenic proteins.

**Method:**

A multicenter, triple-blinded, randomized, crossover prospective clinical study was conducted in dogs suspected to have cutaneous adverse food reaction. The study utilized a hydrolyzed salmon (HS) diet and a hydrolyzed poultry feather (HPF) diet in separate elimination diet trials to determine if the HS diet would be efficacious and well-tolerated, compared with the established HPF diet, to diagnose and treat CAFR.

**Results:**

Fifty-seven dogs were enrolled, and 47 dogs completed the study. HS was well-tolerated, similar to HPF. Pruritus scores during the initial elimination diet trial were reduced with both diets, and dermatitis severity scores during both diet trials were reduced with both diets in the 47 dogs diagnosed with either CAFR, CAFR with atopic dermatitis (AD), or AD. Over half of the subjects diagnosed with CAFR or CAFR with AD required >4 weeks to show PVAS score decreases ≥2 or any decrease in CADESI-4 score.

**Discussion:**

HS, like HPF, presents a valuable diagnostic and treatment tool for dogs suffering from CAFR. Both hydrolyzed diets tested also improved clinical signs in dogs diagnosed with AD and may be useful adjunctive tools in the management of canine AD.

## 1 Introduction

Canine cutaneous adverse food reaction (CAFR) is a common disorder caused by abnormal and unwanted skin reactions to ingested dietary allergens (1, 2). Whereas other forms of allergic dermatitis may require drug therapy, CAFR is best treated with dietary change. Therefore, early and accurate diagnosis and treatment of CAFR are critical.

The gold standard test for CAFR is a 6–12 week elimination diet trial (EDT) using limited and hypoallergenic (novel or hydrolyzed) protein ingredients (3, 4). Relapse of clinical signs upon dietary provocation challenge (i.e., re-introduction of previously fed ingredients) at the end of an EDT confirms CAFR (4). Novel protein diets rely on reduced allergenicity due to lack of prior exposure to the protein allergen; hydrolyzed diets rely on reduced allergenicity through hydrolyzation of often commonplace proteins. Hydrolyzed protein diets are important options for EDTs in dogs: it is difficult to source truly novel proteins that will avoid allergic responses in all individuals, and cross-reactions may occur between commonplace and seemingly novel proteins (5). However, hydrolyzed protein diets themselves may fail due to insufficient degree of hydrolyzation of allergenic proteins. Both hypoallergenic diet types may additionally fail due to the presence of unlabeled contaminant ingredients or reactivity to non-protein ingredients within the diets (5). Overall systemic and skin health can be further impacted by dietary ingredients through separate pathways than allergy mediation. Therefore, it is important to test new hypoallergenic diets prior to routine utilization in EDTs. This multicenter, randomized, triple-blinded, crossover, prospective study sought to test the hypothesis that a hydrolyzed salmon diet (HS^1^) would be efficacious and well-tolerated in the diagnosis and treatment of CAFR. The diet was compared with an established hydrolyzed poultry feather diet (HPF^2^).

## 2 Materials and methods

### 2.1 Study design

**Figure 1:**
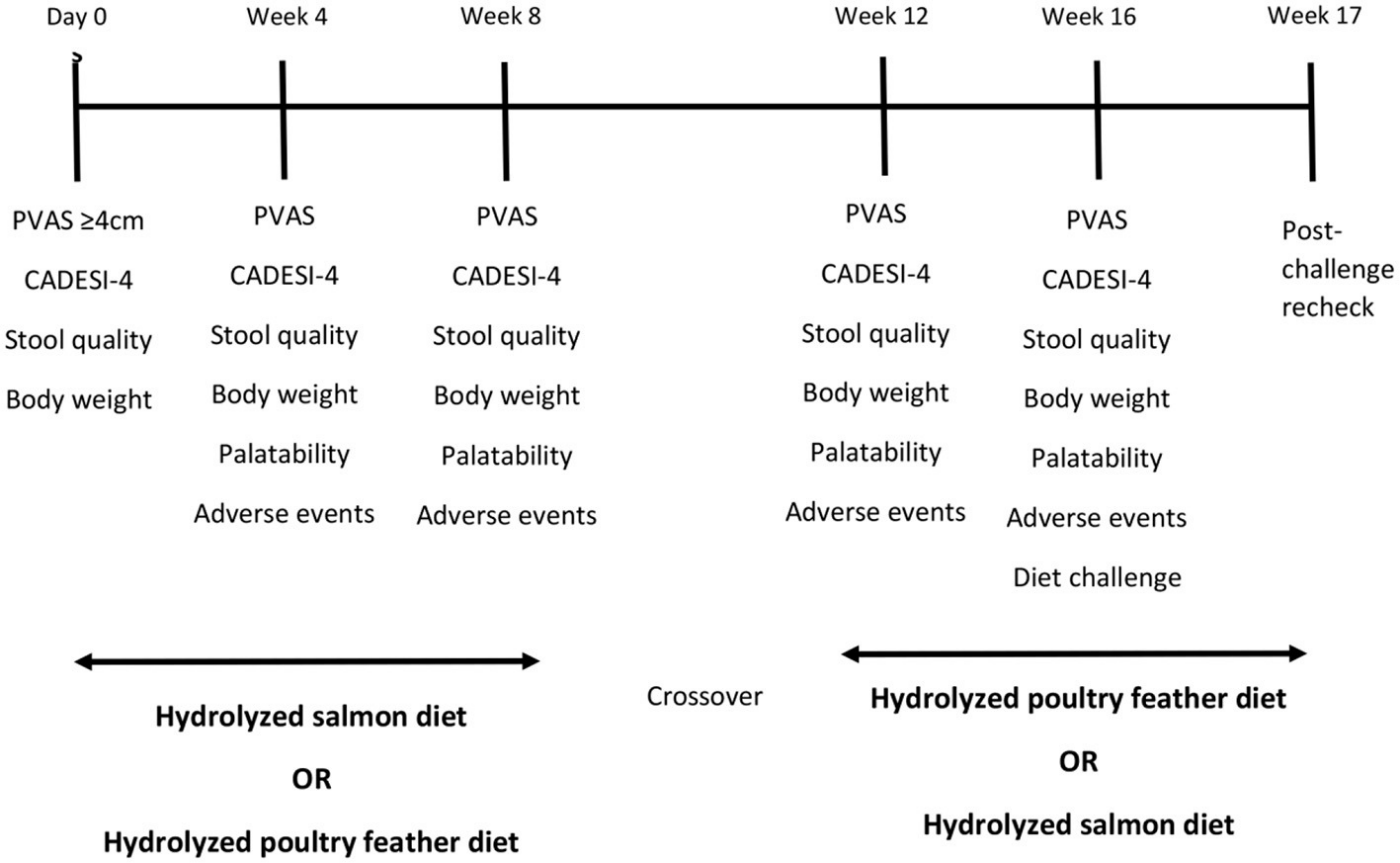
Study design.

This was a multicenter, prospective, triple-blinded, randomized, crossover study to evaluate the efficacy and tolerability of HS and HPF in EDTs. A crossover design was chosen because within-subject variation is less than between-subject variation, lowering the required number of subjects. Dogs suspected by evaluators to have CAFR were enrolled from October 2017 through September 2018 in two consecutive 8-week elimination diet trials (EDT1 and EDT2, respectively). Subjects were randomly assigned by the toss of a coin to one of two treatment sequences: (i) exclusive feeding of HS for EDT1 followed by exclusive feeding of HPF for EDT2, or (ii) HPF first followed by HS. After the end of EDT2, owners of dogs characterized as having CAFR were asked to challenge with their pre-study diet and to schedule an additional examination seven days later, or sooner when flare or exacerbation of clinical signs (e.g., PVAS increase >2) was noted (Figure 1).

Both HS and HPF diets were commercially available, veterinary-exclusive diets indicated for use with dogs having clinical signs of food allergies and in food elimination trials. The diets were extruded kibbles nutritionally complete and balanced for maintenance of adult dogs. Key ingredient and nutrient composition of both diets are shown in Table 1.

**Table 1:** Key nutrient and ingredient composition of study diets.

	**Hydrolyzed salmon diet**	**Hydrolyzed poultry feather diet^*a*^**
**Nutrient (g/100 kcal)**		
Protein	7.19	4.66
Fat	3.69	4.28
Carbohydrate	12.34	12.26
Crude fiber	1.03	0.57
Total dietary fiber	2.48	1.56
Omega-3 fatty acids	0.55	0.16
**Metabolizable energy**		
(kcal/cup)	385	313
(kcal/kg)	3569	3688
**Ingredients**		
	Salmon hydrolysate^*b*^	Corn starch
	Pea starch	Hydrolyzed poultry by-products aggregate
	Potatoes	Coconut oil
	Peas	Soybean oil
	Pea protein	Natural flavors
	Canola oil	Potassium phosphate
	Potato starch	Powdered cellulose
	Natural flavor	Calcium carbonate
	Pea fiber	Sodium silico aluminate
	Flaxseed	Chicory
	Calcium carbonate, dicalcium phosphate, pumpkin, dried kelp, fish oil, dehydrated alfalfa meal, dried chicory root, salt, choline chloride, caramel color, vitamins, DL-methionine, mixed tocopherols, L-tryptophan, parsley, blueberries, cranberries, barley grass, yucca schidigera extract, turmeric, oil of rosemary, L-carnitine, L-lysine, trace minerals, taurine, dried yeast, dried *Enterococcus faecium* fermentation product, dried *Lactobacillus acidophilus* fermentation product, dried *Aspergillus niger* fermentation extract, dried *Trichoderma longibrachiatum* fermentation extract, dried *Bacillus subtilis* fermentation extract	L-tyrosine, fructooligosaccharides, fish oil, L-lysine, choline chloride, taurine, L-tryptophan, vitamins, D-L methionine, marigold extract, histidine, trace minerals, rosemary extract, mixed tocopherols, citric acid

Information from BLUE Natural Veterinary Diet 2018 and Royal Canin Veterinary Diet 2017 product guides.

^a^Mean molecular weight of diet <1,000 Da.

^b^Mean molecular weight of salmon hydrolysate, 2,000 Da.

All evaluators were board-certified or residency-trained veterinary dermatologists. Blinding of owners, evaluators, and the statistician was maintained: diets were dispensed in sealed, white bags labeled only with a letter A or B (HPF or HS, respectively); and data pertaining to subject and diet identification were held by an individual within each practice whose only role in the study was randomization and food dispensation. The HS diet was provided by the manufacturer in sealed white bags. The HPF diet was transferred to the same resealable white bags at a single time by the designated individual within each practice, taking care to reduce the risk of contamination. For each diet, all product dispensed for use originated from the same manufacturer's lot.

### 2.2 Selection of study subjects

An *a priori* power analysis established that 50 subjects would be needed to achieve a power >80% of detecting between diets, in each dog, a minimum difference of 1 cm (± 2.4 cm) in the Pruritus Visual Analog Scale (PVAS) score, with an alpha error rate of <0.05 using a two-tailed paired *t*-test. The use of PVAS score to evaluate response to diet has been previously reported (5–7). Subjects were client-owned and selected from six private-practice dermatology specialty clinics located in the Southwestern and Western United States. Written informed consent was obtained from the dog owners, who were advised that they could withdraw their dog from the study at any point for any reason. Subjects were selected by the following inclusion criteria: (i) suspicion of CAFR based on compatible historical and cutaneous clinical signs, as well as exclusion of ectoparasitic and endoparasitic infestations and cutaneous infections; (ii) perennial pruritic dermatitis of minimum 1-year duration; (ii) PVAS score ≥4 cm, consistent with mild or greater pruritus; and (iii) an adult dog (i.e., growth phase completed). Previously established diagnosis of CAFR was not a prerequisite. Subjects were excluded based on the following: (i) exclusively seasonal pruritus; (ii) non-food related gastrointestinal inflammation and/or internal parasitic disease; (iii) exclusively non-cutaneous manifestations of food allergy; (iv) concurrent secondary infections (bacterial or *Malassezia* spp.) or ectoparasitic infestations; (v) concurrent medical disorder requiring chewable medications; (vi) concurrent medical disorder requiring a therapeutic diet.

At enrollment, subjects had been withdrawn from immunomodulatory therapies: lokivetmab^3^ or injectable long-acting glucocorticoids for minimum 8 weeks; short-acting glucocorticoids or cyclosporine modified for minimum 4 weeks; and oclacitinib,^4^ supplemental fatty acids, or antihistamines for minimum 2 weeks. Withdrawal times were based on published protocols and pharmacokinetic data (8–10). Intestinal parasite screening was performed by fecal ova/parasite flotations and enzyme-linked immunosorbent assay for *Giardia* spp.^5^ For the study duration, subjects were required to take monthly non-oral or non-flavored oral heartworm prophylaxis, and topical flea control was required in flea endemic areas.

### 2.3 Interventions

The EDT1 was initiated on Day 0; EDT2 at Week 8. For humane purposes, subjects could be administered oclacitinib orally q24hr for up to 3 weeks after new diet initiation if needed, with a mandatory seven-day withdrawal prior to examinations. Systemic or topical glucocorticoids, supplemental fatty acids, antihistamines, lokivetmab, or cyclosporine modified were not allowed throughout the study. Antimicrobial baths, sprays, leave-on rinses, wipes, or mousses were permitted. In addition, all owners were advised to discontinue treats, supplements, flavored medications, and toothpastes for the duration of the study.

Each EDT could be prematurely discontinued if there was a pruritic flare or other unacceptable adverse event. Development of pyoderma, *Malassezia* dermatitis, or otitis externa between weeks 4 and 8 of either EDT was interpreted as both an adverse event and as lack of diet efficacy, substantiating immediate discontinuation of that diet. This prompted either prematurely crossing over to the second diet (EDT2) from EDT1 or prematurely ending the study if the subject was already in EDT2. Infected subjects were treated with topical antimicrobials as listed above unless the study was ended, in which case systemic treatment could be initiated. If a subject did not tolerate or accept a diet, that diet was prematurely discontinued, again resulting in either crossing over to EDT2 or ending the study if the subject was already in EDT2.

At the beginning of EDT1, owners were directed to feed the same quantity of food with the study diet as before study enrollment. Feeding amounts were then adjusted at each examination during EDT1 and EDT2 as needed, based on changes in the subject's weight. No diet transition period was included at the beginning of EDT1 or between EDT1 and EDT2.

### 2.4 Outcome assessments

Subjects were examined at enrollment (Day 0) and every 4 weeks, for a possible total of five visits. The primary outcomes measured were changes in PVAS score at weeks 4 and 8 during each EDT and after provocation dietary challenge. Owners were asked to score pruritus using PVAS on Day 0 and every subsequent examination (5 possible timepoints). Both the absolute PVAS scores and the degree of change of PVAS scores were evaluated.

Secondary outcomes assessed were dermatologic lesions and tolerability of each diet. Evaluators assessed dermatologic lesions with the Canine Atopic Dermatitis Extent and Severity Index (CADESI)-4 on Day 0 and at every subsequent examination (5 possible timepoints). Changes in CADESI-4 score were compared both between diets and between diagnosis groups (CAFR, atopic dermatitis [AD], or both). Tolerability also was assessed at each examination through stool quality, changes in body weight, diet acceptance, and diet-related adverse reactions. Owners were asked to report stool quality at each examination using a visual scoring guide,^6^ as well as any non-stool-related adverse events, including anorexia/reluctance to eat diet (from which the authors inferred acceptability).

Evaluators diagnosed subjects with CAFR if PVAS score decreased ≥2 cm after 8 weeks on either or both diet(s), based on previous evidence for indication of adequate therapeutic response (10). A subject was diagnosed with CAFR and closed out of the study if it improved with PVAS score decrease ≥2 in EDT1 and flared with pruritus within 1 week of starting EDT2, suggesting the second diet acted as a provocation dietary challenge. Subjects were diagnosed with AD if the PVAS score decreased <2 in response to either diet, in addition to negative findings for other causes of pruritus. Subjects were diagnosed with both CAFR and AD if one of the following was noted: (i) PVAS decreased ≥2 during either EDT, there was a flare with provocation dietary challenge, and the subject had a history of seasonal pruritus superimposed on non-seasonal pruritus; or (ii) PVAS score decreased ≥2 and there was a flare with provocation dietary challenge, but ongoing absolute PVAS score remained >1.9 after either/both EDT(s). A previous study demonstrated that PVAS score >1.9 is abnormal (11). Therefore, evaluators characterized the subjects in the latter group as having sufficient improvement (PVAS score decrease ≥2) to be diagnosed with CAFR but having higher than normal post-EDT levels of pruritus (final PVAS score >1.9), which is consistent with concurrent AD. Changes in PVAS scores were compared between diets and between diagnosis groups (CAFR, AD, or both).

### 2.5 Statistical analysis

Subject data were entered into a medical records system^7^ and then input into statistical software^8^. Outcome variables of PVAS and CADESI-4 scores were assessed in separate mixed linear models with the subject as a random effect and diet and diagnosis group as fixed effects. Autoregressive covariance structures were used. As clinical scores between diets were not significantly different, statistical equivalence testing was performed. Anderson and Hauck's test and Schuirmann's two one-sided tests were used to assess bioequivalence (defined as ±20%). Proportional distributions of adverse events between diets were evaluated with McNemar's exact test for matched/paired binary data. Summary statistics are expressed as median and range for non-parametrically distributed data (age, weight, PVAS and CADESI-4 scores) and as mean ± standard deviation (SD) for parametrically distributed data (changes in PVAS and CADESI-4 scores over time). Fecal scores are reported as mean ± SD to quantify small changes in ordinal data. Statistical significance was set as *P* < 0.05.

## 3 Results

**Figure 2:**
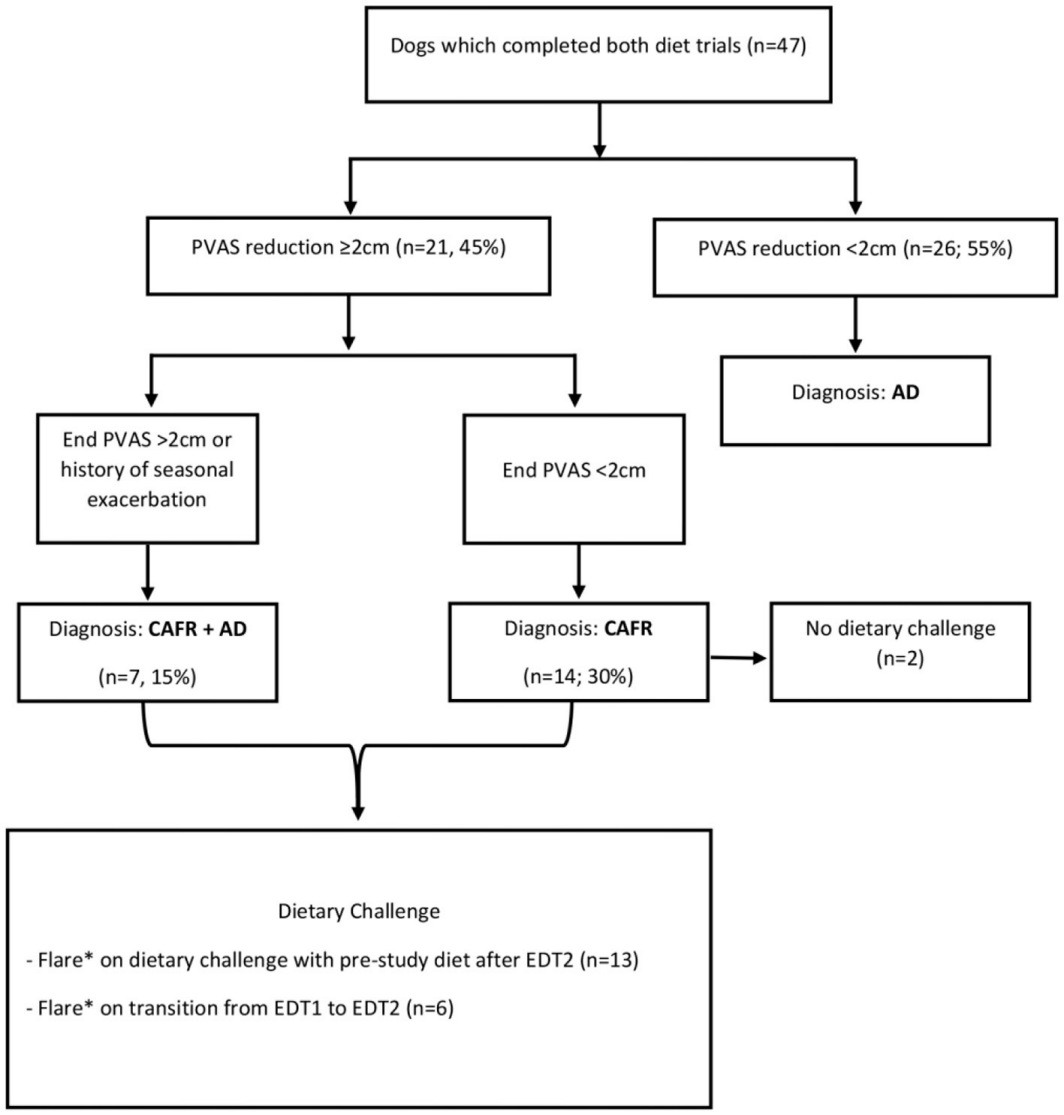
Results: diagnosis.

Fifty-seven dogs that varied in age, sex, size, and breed were enrolled (Table 2). Forty dogs (70.2%) were enrolled by two of the six sites. Of the 57 enrolled, 47 dogs (82.4%) completed both EDT1 and EDT2, resulting in a diagnosis of CAFR, CAFR+AD, or AD (Figure 2). Of the 10 dogs that did not complete the study, 3 were dropped because the owner did not return calls, 6 were voluntarily withdrawn by the owner, and 1 moved. Unless otherwise indicated, all analyses will refer to the 47 dogs that completed both EDTs.

**Table 2:** Baseline demographics.

**Characteristics**	
**Sex**	**No. of dogs (% of total)**
Male intact (%)	1 (1.7%)
Male neutered (%)	31 (54.4%)
Female intact (%)	0 (0%)
Female spayed (%)	25 (43.9%)
**Weight & Age**	**Median (range)**
Weight (kg)	21.9 (2.3–47.0)
Age (years)	3.3 (1.0–12.1)
**Breed**	**No. of dogs**
Mixed breed—small (<10 kg)	11
Mixed breed—large (>10 kg)	10
Labrador retriever	5
German shepherd	5
Pit bull	4
English bulldog	3
Golden retriever	2
Australian cattle dog, Australian shepherd, Australian terrier, boxer, Catahoula leopard dog, chihuahua, cocker spaniel, lhasa ahpso, miniature schnauzer, miniature pinscher, Plot hound, Portuguese water dog, pumi, rat terrier, West Highland white terrier, wire-haired pointing griffon, Yorkshire terrier	1 of each breed

### 3.1 Assessment of pruritus

During EDT1 (Day 0 to week 8), PVAS scores significantly reduced over time (−1.6 ± 2.2; *P* ≤ 0.001), and reductions in PVAS score during EDT1 significantly differed by diagnosis (*P* < 0.001): mean reductions were greatest in dogs with CAFR (−3.4 ± 1.7; *P* < 0.0001), followed by both CAFR and AD (−2.1 ± 2.1; *P* = 0.2999), and finally AD (−0.8 ± 1.7; *P* = 1.0). There was no significant difference in mean PVAS score reduction between HPF and HS (−1.7 ± 2.0 and −1.8 ± 2.2, respectively; *P* = 0.325; Table 3).

**Table 3:** Mean PVAS and CADESI-4 scores by diet.

**Diet**	**Pruritus visual analog score**				**Canine atopic dermatitis extent and severity index-4 (range, 0–180)**			
	**Baseline (range)**	**Reduction in EDT1 (**±**SD)**	**Reduction in EDT2 (**±**SD)**	**Mean Reduction (**±**SD)**	**Baseline (range)**	**Reduction in EDT1 (**±**SD)**	**Reduction in EDT2 (**±**SD)**	**Mean Reduction (**±**SD)**
Hydrolyzed Salmon (*n =* 31)	7.4 (4.5–9.5)	−1.8 ± 2.2	−0.1 ± 2.6	1.1 ± 2.5	32 (range, 8-104)	−8.4 ± 13.3	−5.4 ± 8.2	−7.1 ± 11.4
Hydrolyzed Poultry Feather (*n =* 26)	7.5 (5.0–9.7)	−1.7 ± 2.0	−0.2 ± 2.9	−0.8 ± 2.5	27.5 (range, 7–67)	−6.5 ± 9.9	−0.7 ± 11.8	−3.1 ± 11.4
***P*** **-value**	0.516	0.325	0.580	ND	0.165	0.593	0.131	ND

ND, Not done.

In contrast, during EDT2 (weeks 8–16), PVAS scores did not significantly reduce over time (−0.6 ± 2.6; *P* = 0.169), and changes in PVAS scores did not significantly differ by diagnosis (*P* = 0.884): mean changes were 0.1 ± 3.8 for CAFR, −0.5 ± 2.5 for both CAFR and AD, and −0.2 ± 2.1 for AD (all *P* = 1.0). Similar to EDT1, there was no significant difference in mean PVAS score changes between HPF and HS (−0.2 ± 2.9 and −0.1 + 2.6, respectively; *P* = 0.580; Table 3).

**Figure 3:**
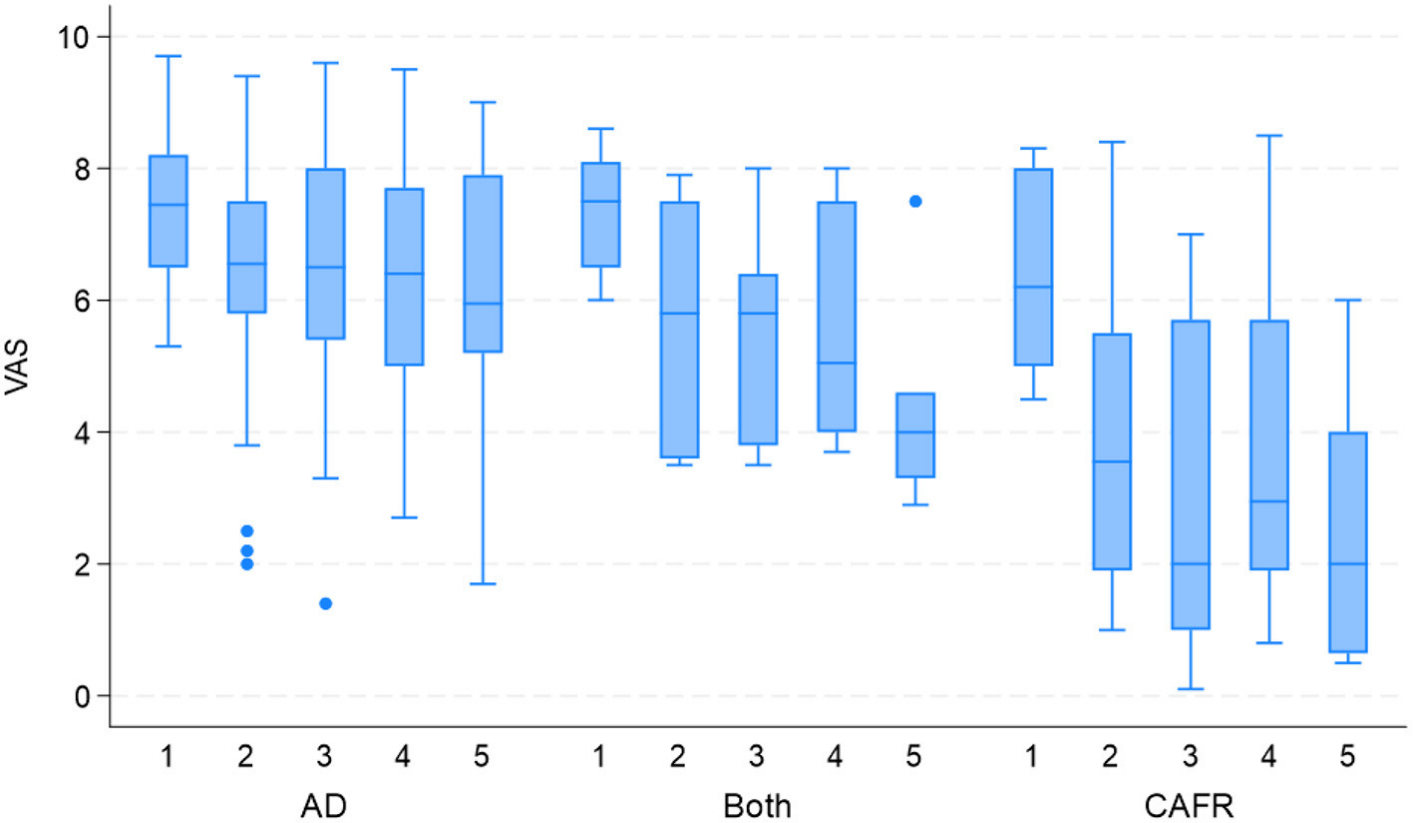
PVAS box-and-whiskers plot for subjects with AD, CAFR, and both CAFR and AD at visits 1 (day 0), 2 (week 4), 3 (week 8), 4 (week 12) and 5 (week 16).

Figure 3 shows PVAS scores at each examination for both EDTs by diagnosis. Of the 14 subjects ultimately diagnosed with CAFR with post-EDT diet challenge, four (28.6%) required >4 weeks to attain a PVAS score reduction ≥2. During the first 4 weeks of the EDT, one of these four dogs showed only a mild PVAS score reduction (−0.3), while another showed a slight increase in PVAS score (+0.4). These two dogs required 4–8 weeks of the EDT to demonstrate a more substantial PVAS score reduction (2.7 and 5.9, respectively). Similarly, of the seven dogs diagnosed with both CAFR and AD, three (42.9%) required weeks 4–8 of the EDT to demonstrate a PVAS score reduction ≥2. Therefore, of the 21 dogs diagnosed with CAFR or CAFR and AD, only 10 dogs (47.6%) had a PVAS score reduction ≥2 by week 4 of the EDT.

### 3.2 Assessment of CADESI-4

During EDT1 (Day 0 to week 8), CADESI-4 scores significantly reduced over time (−8.3 ± 11.8; *P* = 0.001), and mean reductions in CADESI-4 scores did not significantly differ by diagnosis. Mean reductions were −10.2 ± 11.7 for CAFR (*P* = 0.0258), −9.4 ± 6.1 for both CAFR and AD (*P* = 0.9397), and −5.7 ± 13.2 for AD (*P* = 0.6327). There was no significant difference in CADESI-4 score reduction between HPF and HS (−6.5 ± 9.9 and −8.4 ± 13.3, respectively; *P* = 0.593; Table 3).

Similarly, during EDT2 (weeks 8–16), CADESI-4 scores were significantly reduced over time (−3.7 ± 9.9; *P* = 0.038), and mean reductions in CADESI-4 scores did not significantly differ by diagnosis (*P* = 0.789). Mean reductions were −3.4 ± 5.3 for CAFR, −0.1 ± 12.0 for both CAFR and AD, and −3.0± 12.4 for AD (all *P* = 1.0). As above, there was no significant difference in CADESI-4 score reduction between HPF and HS (−0.7 ± 11.8 and −5.4 ± 8.2, respectively; *P* = 0.131; Table 3).

As score outcomes did not significantly differ between diets, equivalence testing between diets was performed, but all tests failed to reject the null hypothesis of >20% difference (*P* ≥ 0.113).

**Figure 4:**
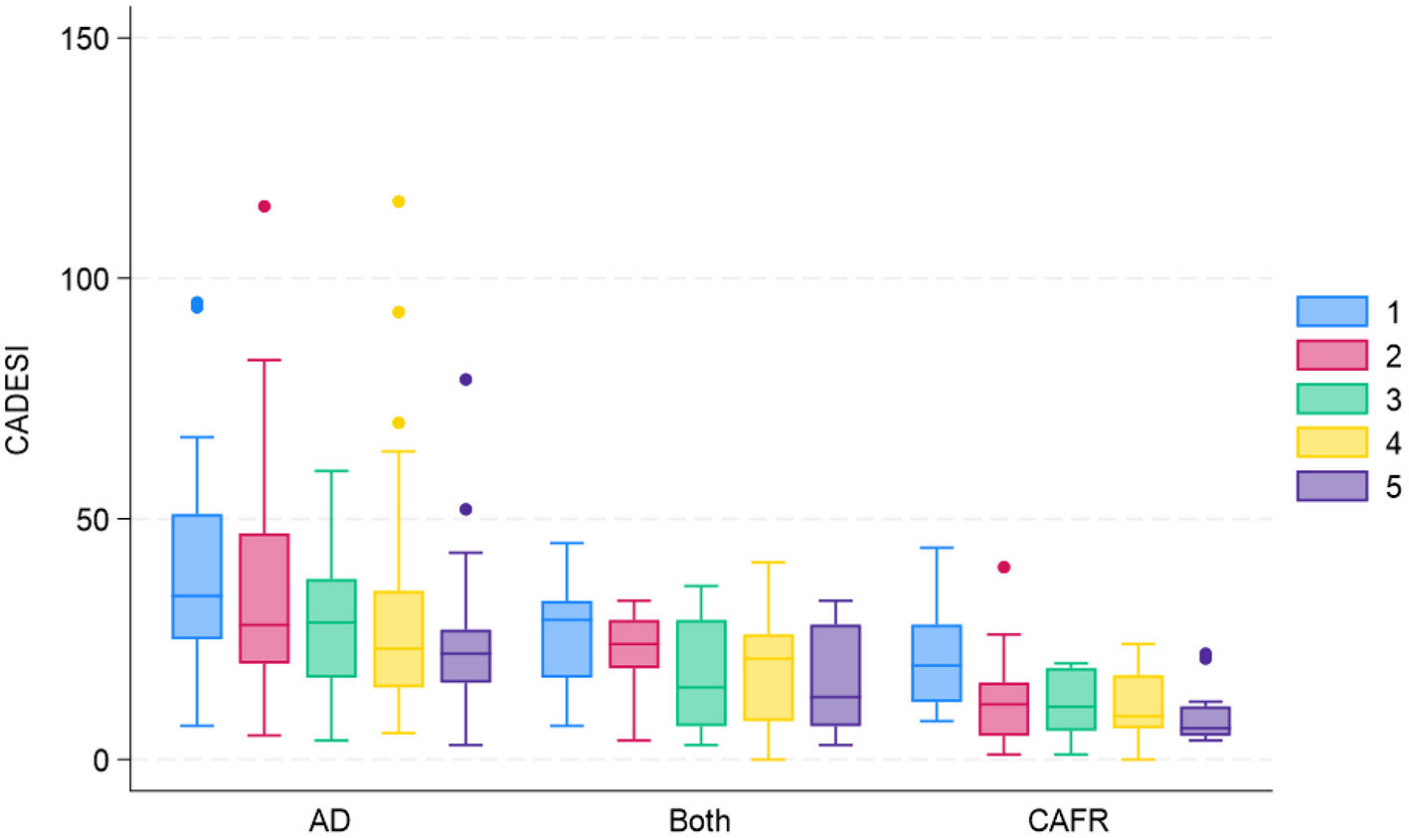
CADESI-4 box-and-whiskers plot for subjects with AD, CAFR, and both CAFR and AD at visits 1 (day 0), 2 (week 4), 3 (week 8), 4 (week 12) and 5 (week 16).

Figure 4 shows CADESI-4 scores at each examination for both EDTs by diagnosis. Of the 14 subjects diagnosed with CAFR, eight (57.1%) required >4 weeks to show any reduction in CADESI-4 score on either diet. Similarly, of the seven dogs diagnosed with both CAFR and AD, three (42.8%) required >4 weeks to demonstrate a CADESI-4 score reduction on either diet. In 4/21 (19%) dogs, the CADESI-4 score increased during the first 4 weeks and then decreased by the 8-week evaluation; 3 of the 4 dogs had a diagnosis of CAFR only. Therefore, of the 21 subjects diagnosed with CAFR or CAFR and AD, only 10 (47.6%) showed CADESI-4 score reduction by week 4 of the EDT.

### 3.3 Assessment of stool quality and body weight

All 57 enrolled dogs (including those prematurely removed from the study) were included in fecal score and body weight analysis. Fecal scores prior to EDT1 ranged from 3 to 5, with nearly all baseline scores of 3 or 4. Fecal scores were significantly greater (i.e., softer stools) on HPF (4.2 ± 0.6) than HS (3.7 ± 0.6) (*P* < 0.001). No dog had diarrhea (fecal score 6 or 7). Owners reported soft stools (fecal score 4 or 5) in 21/57 (36.8%) subjects: two had soft stools on HS but normal stools on HPF; 15 had soft stools on HPF but normal stools on HS; and four had soft stools on both diets. One subject on HS had excessively hard stool (fecal score 1) reported at week 4 but a reported fecal score 4 at all other examinations. Although no owners reported hard stools on HPF, owners of 2 dogs that completed the study reported constipation while on that diet (infrequent or difficult evacuation).

Weight loss occurred with both diets but was not significantly different for HS compared to HPF (−0.35 ± 1.04 kg vs. −0.13 ± 1.20 kg, respectively) (*P* = 0.307). No owner reported concern about weight loss.

### 3.4 Adverse events

All 57 enrolled subjects (including those that prematurely discontinued the study) were included in the analysis of adverse events (Table 4). Adverse events were not significantly different between the diets for either the number of subjects affected (15 with HPF vs. seven with HS, *P* = 0.088) or the total number of adverse events (17 with HPF vs. eight with HS, *P* = 0.072). Overall, 68% of the adverse events were reported when dogs were fed HPF.

**Table 4:** Adverse events by diet.

**Adverse event**	**Number of adverse events by diet**	
	**Hydrolyzed salmon**	**Hydrolyzed poultry feather**
Anorexia/hyporexia	2	3
Borborygmi	0	2
Pruritus	2	6
Pyoderma/otitis	1	3
Vomiting	3	3
Total (% of all events)	8 (32%)	17 (68%)

### 3.5 Owner overall satisfaction

Of the 21 subjects diagnosed with some component of CAFR, owners of 10 chose to feed HS on a long-term basis, while five were maintained on HPF. Owners of the remaining six subjects elected to feed over-the-counter “novel protein” alternatives.

## 4 Discussion

In this multicenter, randomized, triple-blinded, crossover, prospective clinical trial, we hypothesized that HS would be efficacious and well-tolerated for use in EDTs to diagnose and treat CAFR. HS was associated with significant reductions in PVAS and CADESI-4 scores, supporting our hypothesis.

### 4.1 Diet efficacy

Interestingly, for both HS and HPF, PVAS and CADESI-4 scores were reduced not only in subjects ultimately diagnosed with CAFR or CAFR with AD, as one might expect, but also in subjects with AD alone. Unsurprisingly, PVAS score reductions with the diets were most significant in dogs with CAFR, followed by dogs with both CAFR and AD. However, dogs with AD did have a numerical decrease in PVAS score, and CADESI-4 score reductions were not significantly different between subjects with any diagnosis. In other words, even dogs with AD experienced a decrease in pruritus and skin lesion scores on either diet. Therefore, these diets may present useful adjunctive tools in the management of canine AD.

Several factors contributed to PVAS and CADESI-4 score reductions in dogs with AD in this study. Some AD dogs in this study may have benefited from changes in environmental allergen exposure during the multi-week EDTs (e.g., most likely due to season change; less likely due to physical relocation or travel not declared to study personnel), reinforcing the need to confirm flare upon a provocation dietary challenge to definitively diagnose CAFR in clinical practice. Additionally, some subjects diagnosed with AD could have had a component of CAFR but did not have a PVAS decrease ≥2 cm, which was used by this study to diagnose CAFR. This was suspected in several subjects by both the evaluators and owners, based on history of clinical signs and results of physical examination and preliminary diagnostics. Finally, some dogs with AD, without any component of CAFR, will improve during diet trials (12, 13). One proposed reason has been dietary essential fatty acids (EFAs), for which the mechanisms of action in AD have not been fully elucidated but likely include reductions in inflammatory cell activation, alterations in eicosanoid production, and improvements in skin barrier (14). Both HS and HPF included measurable amounts of omega-3 fatty acids (0.55 vs. 0.16 g/100 kcal; Table 1), which may have diminished clinical signs of inflammation. Clinical effects of EFAs in canine AD may take 4–12 weeks; therefore, the 16-week duration of this study could have provided sufficient time to see these effects (14). Lastly, the administration of oclacitinib, although temporary, may have contributed to the observed score improvements in dogs with AD.

In addition, the variation in diet composition between HS and HPF, including protein and omega-3 content (Table 1), may have contributed to subtle but physiologically significant differences in improvement in dermatologic lesions and skin condition assessed in CADESI-4 scoring. In fact, the overall mean CADESI-4 score reduction when dogs were fed HS (−7.1 ± 11.4) was more than double than when fed HPF (−3.1 ± 11.4; Table 3), which may be attributable to HS fulfilling the recommended 25-30% dry matter protein content for dogs with nutrient-responsive dermatologic diseases (15). Although this difference in CADESI-4 score reduction did not meet the established *P*-value cutoff for significance, a recent publication suggests clinical significance and statistical significance may not always be correlated (16).

### 4.2 EDT duration

Clinical practices need a full 8-week or longer EDT, as emphasized by an important finding in this study. Over half of the subjects diagnosed with CAFR or CAFR with AD required >4 weeks to show PVAS score decreases ≥2 or any decrease at all in CADESI-4 score. In 19% of those subjects, the CADESI-4 score actually increased during the first 4 weeks and then decreased, and this was also seen with PVAS in one dog.

### 4.3 Diet tolerability

Both HS and HPF were well-tolerated, as determined by body weight changes, diet acceptance (inferred from owner-reported anorexia/hyporexia), and adverse events. More than 80% of the dogs originally enrolled in the study completed the protocol, and most owners of subjects diagnosed with CAFR elected to remain on the hypoallergenic diet long-term, suggesting good compliance and overall satisfaction with the diets.

Subjects lost weight on both HS and HPF with no significant difference between the two diets. Importantly, no study subject became underweight, and no owner reported concern about weight loss. Anorexia/hyporexia were reported in only two subjects with HS and three subjects with HPF, suggesting both diets were acceptable to most dogs and that diet acceptance was not a major factor in the observed weight loss. Several additional factors could explain a reduced caloric intake resulting in unintended weight loss. Since owners were not instructed to feed the diets per the manufacturer's specific feeding guidelines for weight maintenance, to maintain blinding of owners and clinic staff, subjects may have been offered fewer calories than they received with the pre-study diets. In addition, owners were instructed to feed only the study diets, without extra calories from treats or other dietary additions. Although there was no significant difference between HS and HPF in the number of subjects experiencing adverse events or the total number of adverse events, most of these events were reported when the dogs were fed HPF (17/25 events, 68%). As indicated above, the difference between clinical vs. statistical significance is becoming increasingly recognized in veterinary medicine (16), and a larger study population may have enabled statistical significance of adverse events. The most common adverse event was pruritus (6 events with HPF vs. 2 events with HS). Continued pruritus, anorexia/hyporexia, borborygmi, pyoderma, otitis, and vomiting are of clinical importance for veterinarians and pet owners, and their prevalence should be considered when making diet recommendations.

HPF was associated with significantly softer stools than HS, though no subject had overt diarrhea. Overall, this is a softer stool quality for HPF than was previously reported (5). In that study, HPF was associated with diarrhea in one out of 10 dogs studied, and the remainder had a stool consistency of two (i.e., normal). Hydrolyzed diets may cause soft stools or diarrhea, which is generally related to osmolarity and the degree of hydrolysis and may be moderated by addition of dietary fiber (5, 9).

Both HS and HPF are indicated for use with dogs having clinical signs of food allergies and in food elimination trials, but their ingredient and nutrient composition differ considerably. These differences would reasonably be expected to affect the response to the diet and may have contributed to variation in observed endpoints. For example, the higher omega-3 fatty acid content in the HS diet may have contributed to the lower numerical prevalence of pruritus as an adverse event. Similarly, the difference in fiber content and sources between the diets could have influenced stool quality and the prevalence of soft stool/diarrhea as an adverse effect. These physiological effects and clinical findings, as well as owner preference for the diet after the study, support the indication of HS for nutritional management of dogs with CAFR and AD.

## 5 Limitations

As the diagnosis of CAFR was not a prerequisite for study enrollment, each subject's specific allergy was unknown. However, an individual's specific hypersensitivity can play a significant role in response to diet. Twenty to fifty percent of dogs hypersensitive to a parent protein will continue to flare to diets containing its hydrolysate (5). Insufficient hydrolyzation of parent protein ingredients can play a role, as well as cross-reactivity between allergenic proteins, and the presence of allergenic components such as carbohydrate proteins (17–19). Further study of the response to HS of dogs with known hypersensitivities, as well as independent analysis of ingredients for intact/insufficiently hydrolyzed fish protein, is warranted.

The presence of unlabeled dietary ingredients also can affect response to an EDT. An in-depth independent molecular analysis of HS ingredients was outside the scope of this study. However, prior to and separate from this study, the authors sent a sample from a randomly chosen batch of HS to an independent laboratory^9^ for enzyme-linked immunosorbent assay evaluation for unlabeled, intact, common protein contaminants (pork, soy, beef, and poultry). None were found.^10^ A similar analysis has been completed for HPF (20). Further study could include independent testing of multiple random batches of HS. Although transferring the HPF diet into white bags to maintain blinding of owners and staff to diet identity could in theory result in contamination, this process was performed only once at the beginning of the study under clean conditions in an area of the clinic remote from contact with other diets, removing the practical likelihood of introduction of contaminants such as unlabeled dietary proteins, dust mites, or microorganisms.

There was potential for selection bias. All subjects were seen by private practice referral dermatologic clinics, which may have selected for either more readily compliant owners and/or more severely allergic cases. It is not known if this could have contributed to the slightly higher percentage of dogs diagnosed with CAFR in this study population (21/47 dogs, 44.7%) compared with previous studies (2).

There was not a washout (hypoallergenic diet-free) period, which could allow for carryover effect between EDT1 and EDT2. This was not included due to concern that a resulting flare on the washout diet would negatively affect subject well-being, as well as concern that this flare would lead to a larger drop-out rate. This study's crossover design is expected to have statistically reduced the direct impact of the first EDT on the second.

A further potentially controversial aspect of this study was allowing temporary use of oclacitinib. The authors felt justified for several reasons. First, one review estimated that the majority of dogs with CAFR require weeks to achieve improvement in clinical signs after dietary change (3). Given this potential delay in clinical relief, the authors felt the option for temporary initial anti-pruritic therapy was necessary. Additionally, considering the reported short half-life of the drug, the authors felt it unlikely that the use of oclacitinib within the protocol's withdrawal times would impact PVAS scores at the times of evaluation (10). Finally, the authors felt that such administration of oclacitinib would be compatible with clinical practice.

This study's EDT duration was chosen based on previous reports that 90% of dogs with CAFR will respond to an appropriate diet within 8 weeks (3). It is possible that a longer EDT duration would have revealed additional dogs with CAFR. However, one study demonstrated that the use of oclacitinib at the start of an EDT, as was done in this study, may reduce the duration of the EDT needed (19).

It is possible that the 7-day evaluation post-provocation dietary challenge in this study was an insufficient time frame to allow for all subjects to flare. After the design and data acquisition portion of this study was completed, a published review paper reported 50% of dogs will flare after dietary challenge after 5 days, but it may take 14 days to identify flare in others (21). As the primary outcome measurement of this study was PVAS score reduction while on HS or HPF, the authors do not feel that this significantly affected the conclusions of this paper. However, future studies may benefit from a 14-day, rather than 7-day, post-challenge examination.

The total of 47 dogs completing the study was below the power calculation of 50 subjects and likely contributed to lack of statistical significance for some comparisons. It was anticipated that 57 enrollments from both diet groups would be sufficient to yield at least 50 completions, but several subjects dropped out late in the course of the study. Notwithstanding statistical significance, however, findings such as the more than 2-times greater reduction in CADESI scores and less than half the number of adverse events when HS was fed may be considered important in making therapeutic decisions in clinical practice (16).

## 6 Conclusion

In this study, HS was well-tolerated and reduced PVAS and CADESI-4 scores in 47 dogs diagnosed with CAFR, CAFR with AD, or AD, similar to HPF. As such, it presents a valuable diagnostic and treatment tool for dogs suffering from CAFR. A positive response to an EDT may require >4 weeks of feeding, based on the initial lack of reduction in clinical signs in many subjects with CAFR or CAFR with AD in this study. As both HS and HPF also improved clinical signs in dogs diagnosed with AD, these diets additionally may be useful adjunctive tools in the management of canine AD.

## Data Availability

The original contributions presented in the study are included in the article/supplementary material, further inquiries can be directed to the corresponding author.

## Footnotes

^1^BLUE Natural Veterinary Diet™ HF Hydrolyzed for Food Intolerance, Blue Buffalo Co. Ltd., Wilton, CT.

^2^Royal Canin Ultamino, Guelph, Ontario, Canada.

^3^Cytopoint^®^, Zoetis USA.

^4^Apoquel^®^, Zoetis USA.

^5^Idexx Laboratories, Westbrook, ME.

^6^Nestle Purina.

^7^Medrio^®^, San Francisco, CA.

^8^SAS v.9.4, SAS Institute Inc., Cary, NC, USA.

^9^ELISA Technologies, Gainesville, FL.

^10^Unpublished data.
